# Frequency of and Predictive Factors for Vascular Invasion after Radiofrequency Ablation for Hepatocellular Carcinoma

**DOI:** 10.1371/journal.pone.0111662

**Published:** 2014-11-14

**Authors:** Yoshinari Asaoka, Ryosuke Tateishi, Ryo Nakagomi, Mayuko Kondo, Naoto Fujiwara, Tatsuya Minami, Masaya Sato, Koji Uchino, Kenichiro Enooku, Hayato Nakagawa, Yuji Kondo, Shuichiro Shiina, Haruhiko Yoshida, Kazuhiko Koike

**Affiliations:** 1 Department of Gastroenterology, Graduate School of Medicine, The University of Tokyo, Tokyo, Japan; 2 Department of Gastroenterology, Graduate School of Medicine, Juntendo University, Tokyo, Japan; Icahn School of Medicine at Mount Sinai, United States of America

## Abstract

**Background:**

Vascular invasion in patients with hepatocellular carcinoma (HCC) is representative of advanced disease with an extremely poor prognosis. The detailed course of its development has not been fully elucidated.

**Methods:**

We enrolled 1057 consecutive patients with HCC who had been treated with curative intent by radiofrequency ablation (RFA) as an initial therapy from 1999 to 2008 at our department. We analyzed the incidence rate of and predictive factors for vascular invasion. The survival rate after detection of vascular invasion was also analyzed.

**Results:**

During a mean follow-up period of 4.5 years, 6075 nodules including primary and recurrent lesions were treated by RFA. Vascular invasion was observed in 97 patients. The rate of vascular invasion associated with site of original RFA procedure was 0.66% on a nodule basis. The incidence rates of vascular invasion on a patient basis at 1, 3, and 5 years were 1.1%, 5.9%, and 10.4%, respectively. Univariate analysis revealed that tumor size, tumor number, alpha-fetoprotein (AFP), des-gamma-carboxy prothrombin (DCP), and Lens culinaris agglutinin-reactive fraction of alpha-fetoprotein were significant risk predictors of vascular invasion. In multivariate analysis, DCP was the most significant predictor for vascular invasion (compared with a DCP of ≤100 mAu/mL, the hazard ratio was 1.95 when DCP was 101–200 mAu/mL and 3.22 when DCP was >200 mAu/mL). The median survival time after development of vascular invasion was only 6 months.

**Conclusion:**

Vascular invasion occurs during the clinical course of patients initially treated with curative intent. High-risk patients may be identified using tumor markers.

## Introduction

Hepatocellular carcinoma (HCC) is a leading cause of cancer death. It has a particularly high incidence in Asian countries, including Japan [1], [2]. To control this disease, close surveillance using advanced diagnostic modalities including ultrasonography (US), computed tomography (CT), and gadolinium-ethoxybenzyl-diethylenetriamine pentaacetic acid-enhanced magnetic resonance imaging (EOB-MRI) in designated high-risk patients has facilitated HCC detection at a very early stage at which surgical resection, liver transplantation, and percutaneous ablative therapies are feasible [3]. Although surgical resection is usually the first-choice treatment option for early stage disease, it is not frequently indicated in patients with underlying liver function impaired by chronic infection of hepatitis B or C virus [4]. Liver transplantation can treat both cancer and liver dysfunction; it has shown excellent survival rates in patients with early stage HCC [5]. However, in countries where cadaveric donor organs are scarce, as in Japan, the application of liver transplantation is limited.

Radiofrequency ablation (RFA) is currently considered to be the most effective first-line percutaneous ablative therapy because it has greater efficacy in terms of local cure than does ethanol injection [6]. The survival outcomes for patients who achieve a complete response by RFA are comparable with those for patients treated by hepatic resection [7], [8]. However, even after locally curative resection or ablation, patients encounter frequent recurrence in the remnant liver because of intrahepatic spread of tumor cells and metachronous multicentric carcinogenesis; the rate of recurrence at 5 years is as high as 70–80% [9], [10]. Although repeated resection or ablation can be performed in patients with recurrent HCC [11], [12], the tumor tends to be out of control during the clinical course of frequent recurrence and retreatment. This is a major reason for the poor long-term survival after curative resection or ablation [13].

The development of vascular invasion and extrahepatic metastasis are representative events of an advanced stage of HCC [14], [15]. Once tumor cells have invaded the portal vein, they progressively spread and increase the portal venous pressure, resulting in ascites and the rupture of esophageal varices. The spread also decreases portal flow into the hepatic parenchyma, causing fatal liver failure [16]–[18]. Hepatic venous invasion causes tumor thrombi to form in the pulmonary arteries and lung tissue [19], and biliary invasion may cause jaundice, hemobilia, or cholangitis [20]. Previous studies have reported that patients with HCC with vascular invasion survive for only three months [15].

The detailed course of development of vascular invasion could not be fully elucidated by analyzing the cases who unfortunately encountered the advanced disease with vascular invasion at the time of the initial diagnosis. Because patients who undergo RFA are rigorously followed up for recurrence, the use of imaging modalities might allow for the identification of the early form of vascular invasion.

In this paper, we analyzed the incidence and predictive factors of vascular invasion as well as its detailed characteristics in patients with HCC treated with RFA with curative intent as the initial therapy.

## Patients and Methods

### Ethics statement

This retrospective study was conducted according to the ethical guidelines for epidemiological research of the Japanese Ministry of Education, Culture, Sports, Science and Technology and Ministry of Health, Labour and Welfare. The study design was included in a comprehensive protocol at the Department of Gastroenterology, The University of Tokyo Hospital and approved by the University of Tokyo Medical Research Center Ethics Committee (approval number 2058). Informed consent was waived because of the retrospective design. The following statements were posted at a website (http://gastro.m.u-tokyo.ac.jp/med/0602A.htm) and participants who do not agree to the use of their clinical data can claim deletion of them.

Department of Gastroenterology at The University of Tokyo Hospital contains data from our daily practice for the assessment of short-term (treatment success, immediate adverse events etc.) and long-term (late complications, recurrence etc.) outcomes. Obtained data were stored in an encrypted hard disk separated from outside of the hospital. When reporting analyzed data, we protect the anonymity of participants for the sake of privacy protection. If you do not wish the utilization of your data for the clinical study or have any question on the research content, please do not hesitate to make contact with us.

### Patients

From 1999 to 2008, a total of 1057 patients with HCC underwent RFA as the initial treatment for naïve HCC. All the patients were included in this study and followed. The inclusion criteria for RFA were: 1) no prior HCC treatment other than TACE as part of sequential TACE-RFA treatment protocol; 2) three or fewer lesions of ≤3 cm in diameter; 3) a total bilirubin level of <3 mg/dL; 4) a platelet count of ≥50×10^3^/mm^3^; and 5) a prothrombin activity level of ≥50%. Exclusion criteria were: 1) portal vein tumor thrombosis; 2) refractory ascites; or 3) extrahepatic metastasis However, we also performed RFA on patients outside these criteria if treatment was predicted to be clinically effective [21].

### Diagnosis and treatment of primary HCC

HCC was diagnosed using dynamic computed tomography (CT); hyperattenuation in the arterial phase with washout in the late phase was considered to be a definitive sign of this disease [22]. Most nodules were also confirmed histopathologically via ultrasound (US)-guided biopsy. All patients underwent dynamic CT with a slice thickness of 5 mm within 1 month prior to RFA for comparison. The detailed protocol for RFA is described elsewhere [21]. Briefly, a 17-gauge, cooled tip electrode was inserted into the lesion under real-time ultrasound guidance. We started ablation at 60 W for the 3-cm exposed tip and 40 W for the 2-cm exposed tip. The power was increased to 140 W at a rate of 20 W/min. When a rapid increase in impedance was observed during thermal ablation, we minimized the output for 15 seconds and restarted the emission at a lower output. The duration of a single ablation was 12 minutes for the 3-cm electrode and 6 minutes for the 2-cm electrode. During the treatment evaluation, a lesion was judged to be completely ablated when the nonenhanced area shown in the late phase of post-ablation CT covered the entire lesion shown in both the early and late phases of pre-ablation CT with a safety margin in the surrounding liver parenchyma. We confirmed complete ablation in all slices in which the target nodule was visualized. Patients underwent additional sessions until complete ablation was confirmed in each nodule.

### Follow-up and assessment of vascular invasion

The follow-up regimen comprised blood tests to monitor tumor markers in an outpatient setting. Dynamic CT was also performed every 4 months. When HCC recurrence was identified, patients who met the same criteria used for primary HCC underwent RFA. When RFA was not indicated for the recurrent nodules due to their multiplicity, the patient underwent TACE if liver function was categorized as Child-Pugh class B or better. Those with extrahepatic tumor metastasis received systemic chemotherapy if they had well-preserved liver function and a good performance status. Vascular invasion was defined as invasion of an HCC tumor into the first and/or second branch or the main trunk of the vasculature. Vascular invasion was confirmed by demonstrating the following imaging characteristics: 1) a low-attenuation intraluminal mass that expanded the vasculature on CT, MRI, or conventional US[23], [24] or 2) attenuation of portal blood flow and detection of vascularity in the thrombi by contrast-enhanced CT, MRI, or US [25], [26]. The follow-up period was defined as the interval from the date of the initial RFA until the date of diagnosis of vascular invasion development, the date of death, or the end of December 2011.

### Statistical analysis

Cumulative incidence of vascular invasion was calculated using the Kaplan–Meier method. Predictive factors for the development of vascular invasion were analyzed using univariate and multivariate Cox proportional hazard regression. The following factors at the initial therapy were used for the analyses: age, sex, hepatitis B surface antigen positivity, hepatitis C antibody positivity, Child-Pugh class, platelet count, alanine aminotransferase level, maximum tumor size, number of lesions, alpha-fetoprotein (AFP) level, des-gamma-carboxyprothrombin (DCP) level, and *Lens culinaris* agglutinin-reactive fraction of AFP (AFP-L3). In the multivariate analysis, stepwise variable selection based on the Akaike information criterion was used to build the final model. Scatter plots were used to assess the relationship between tumor marker values immediately before the initial treatment and at the time of diagnosis of vascular invasion. We also estimated survival rates after the development of vascular invasion using the Kaplan–Meier method. Survival curves were stratified according to the mode of vascular invasion, which was classified by the level of portal vein invasion. For the survival analysis, the follow-up was censored on 31 December 2012. Differences with a *P* value of <0.05 were considered to be statistically significant. All statistical analyses were performed with R 2.13.0 (http://www.R-project.org).

## Results

### Patient profiles and development of vascular invasion

The enrolled HCC patient cohort in this study comprised 685 males and 372 females with a median age of 68.8 years (Table 1). Approximately 75% of cases were hepatitis C-related. The median [interquartile range (IQR)] maximal tumor size was 2.4 [1.8–3.1] cm. The mean (± standard deviation) number of nodules was 1.7±1.2.

**Table 1:** Patients' characteristics at initial RFA (n = 1057).

Variable	
Age in years	
Median	68.8
IQR	63.4–74.4
Male sex, n (%)	685 (64.8)
Etiology	
HBsAg-positive only, n (%)	119 (11.3)
Anti-HCVAb-positive only, n (%)	789 (74.6)
Both positive, n (%)	11 (1.0)
Both negative, n (%)	138 (13.0)
Alcohol consumption>80 g/day	154 (14.6)
Platelet count (×10^9^/L)	
Median	108
IQR	78–146
Child-Pugh classification, n (%)	
Class A	781 (73.9)
Class B	265 (25.1)
Class C	11 (1.0)
Tumor number, n (%)	
1	622 (58.8)
2–3	350 (33.1)
>3	85 (8.0)
Maximal tumor size (mm)	
Median	24
IQR	18–31
AFP, n (%)	
≤100 ng/mL	832 (78.7)
>100 and ≤200 ng/mL	72 (6.8)
>200 ng/mL	153 (14.5)
DCP, n (%)*	
≤100 mAU/mL	878 (83.6)
>100 and ≤200 mAU/mL	72 (6.9)
>200 mAU/mL	100 (9.5)
AFP-L3, n (%)	
≤10%	878 (83.1)
>10%	179 (16.9)

* Not determined in seven patients due to warfarin use.

Abbreviations: HBsAg, hepatitis B surface antigen; HCVAb, hepatitis C virus antibody; AFP, alpha-fetoprotein; AFP-L3, *Lens culinaris* agglutinin-reactive fraction of AFP; DCP, des-gamma-carboxyprothrombin; IQR, interquartile range.

**Figure 1:**
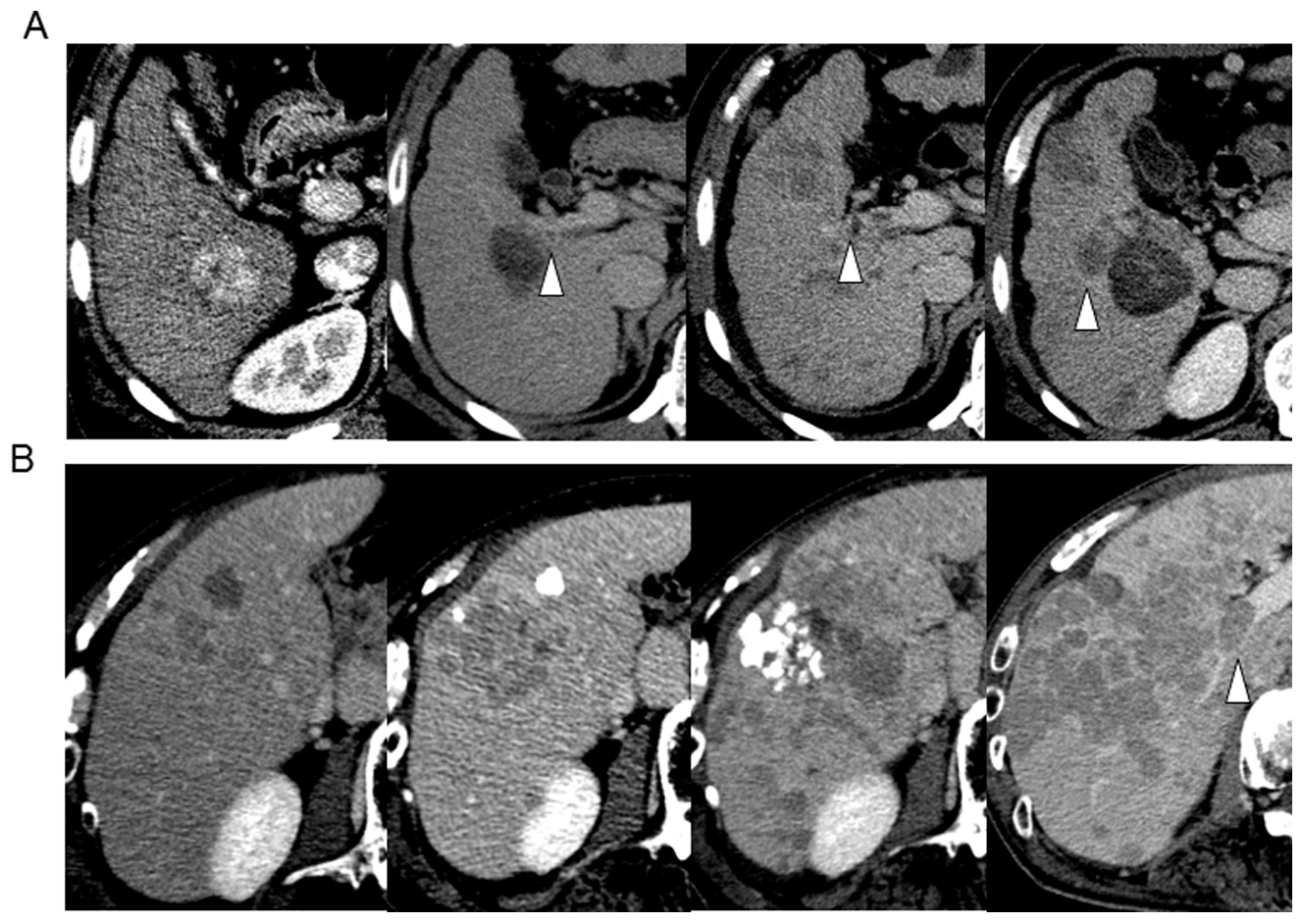
Images of vascular invasion developing adjacent (A) and apart (B) from the ablated area. (Case A: left panel: primary lesion before RFA, middle left panel: development of VI, middle right and right panel: evident development of VI after 4 months, Case B: left panel: multiple recurrence after RFA, middle left panel: after TACE, middle right and right panel: VI development after repeated TACE). Arrowheads denote portal venous invasion.

**Figure 2:**
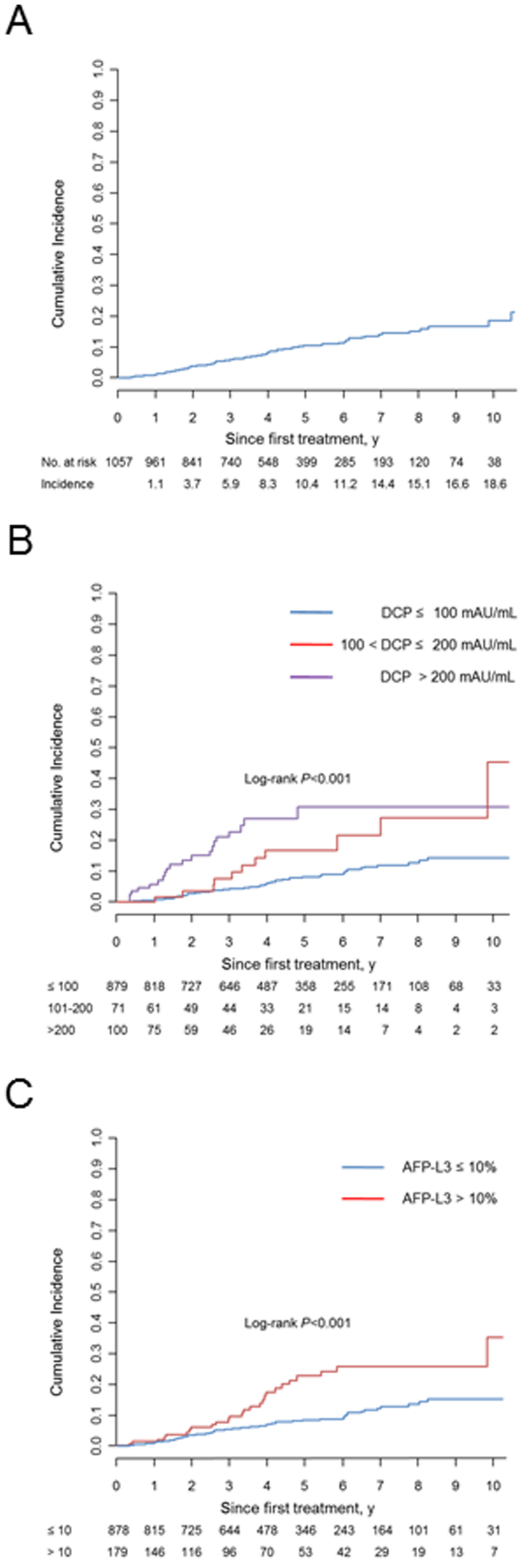
Cumulative incidence of vascular invasion after the initial treatment (A) and incidence stratified based on the DCP (B) and AFP-L3 (C) levels at the initial treatment.

During the mean follow-up period of 4.5 years, 735 of the 1057 enrolled patients underwent 2288 RFA treatments for tumor recurrence in addition to the initial RFA. Thus, the total number of RFA treatments and target nodules were 3345 and 6075, respectively. Vascular invasion was observed in 97 patients, developing adjacent and apart from the ablated area in 40 and 57 patients, respectively (Fig. 1). Therefore, the rate of vascular invasion development associated with site of original RFA was 0.66% on a nodule basis. The detailed tumor characteristics of the patients at the time of diagnosis of vascular invasion are shown in Table 2. The sites of vascular invasion were the portal vein in 85, biliary tract in 17, and hepatic vein in 4. The cumulative incidence rates of vascular invasion on a patient basis at 1, 3, 5, and 10 years were 1.1%, 5.9%, 10.4%, and 18.6%, respectively (Fig. 2A).

**Table 2:** Patients' characteristics at diagnosis of vascular invasion (n = 97).

Variable	
Extension of vascular invasion, n (%)	
Portal invasion within subsegmental branch	20 (20.6)
Portal invasion within segmental branch	22 (22.6)
Portal invasion within first branch	30 (30.9)
Portal invasion to main trunk	12 (12.3)
Bile duct invasion	17 (17.5)
Hepatic venous invasion	4 (4.1)
Tumor location	
Adjacent to previously ablated area, n (%)	40 (41.2)
Apart from previously ablated area, n (%)	57 (58.8)
Tumor number, n (%)	
1	10 (10.3)
2–3	8 (8.3)
4–10	14 (14.4)
>10	44 (45.4)
Undetectable*	21 (21.6)
Maximal tumor size	
Median (mm)	27.5
IQR	15.5–41.0
Diffuse/infiltrative, n (%)	36 (37.1)
Extrahepatic metastasis, n (%)	28 (28.9)
AFP, n (%)	
≤100 ng/mL	34 (35.1)
>100 and ≤200 ng/mL	9 (9.3)
>200 ng/mL	54 (55.7)
DCP, n (%)^†^	
≤100 mAU/mL	31 (32.3)
>100 and ≤200 mAU/mL	10 (10.4)
>200 mAU/mL	55 (57.3)
AFP-L3, n (%)	
≤10%	42 (43.3)
>10%	55 (56.7)

*Intrahepatic tumor was not clearly identified. †DCP was not measured in one patient due to warfarin use.

Abbreviations: HR, hazard ratio; HBsAg, hepatitis B surface antigen; HCVAb, hepatitis C virus antibody; AFP, alpha-fetoprotein; AFP-L3, Lens culinaris agglutinin-reactive fraction of AFP; DCP, des-gamma-carboxyprothrombin.

### Predictive factors related to vascular invasion

**Figure 3:**
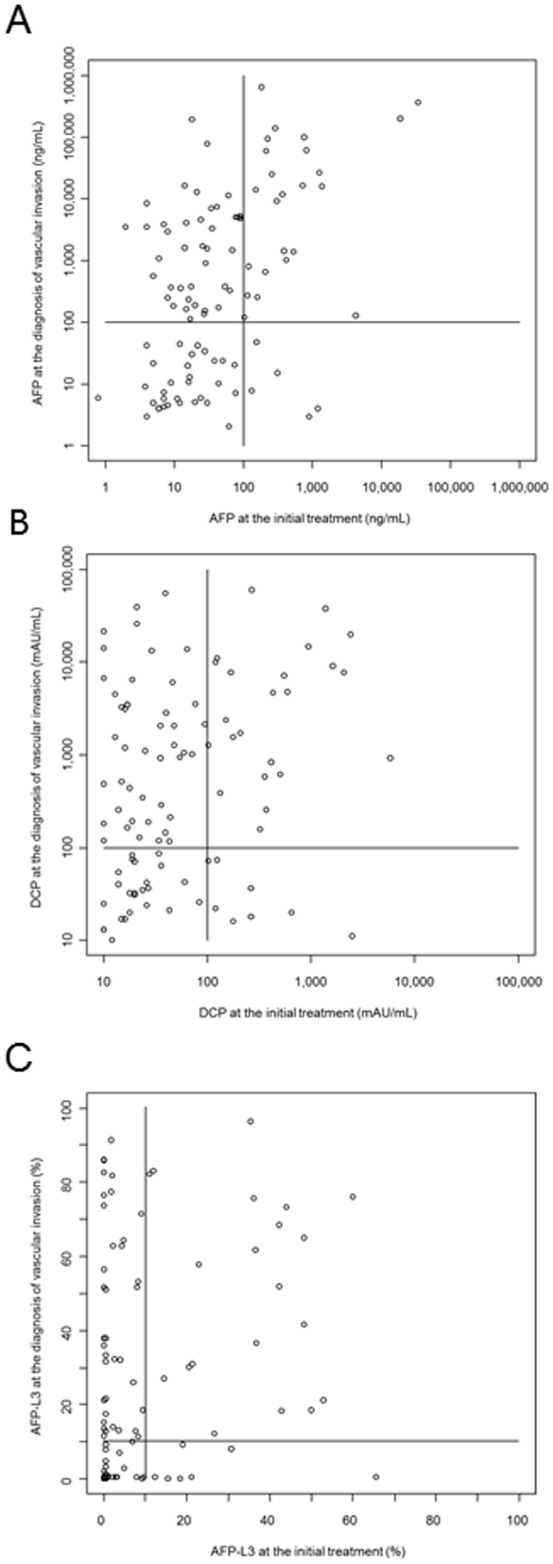
Scatter plots of AFP (A), DCP (B), and AFP-L3 (C) at initial treatment and at diagnosis of vascular invasion.

Univariate Cox proportional regression revealed that the following factors were significantly associated with vascular invasion: tumor size, tumor number, AFP, DCP, and AFP-L3. Multivariate analysis with step-wise variable selection showed that the final model included tumor size, tumor number, DCP, and AFP-L3 (Table 3, Fig. 2B and C). We assessed the relationship of tumor marker values prior to ablation and at the time of diagnosis of vascular invasion. As shown in Figure 3, the sensitivities of tumor markers were higher at the time of diagnosis of vascular invasion than at the time of initial treatment: 64.9% for AFP, 67.7% for DCP, and 47.4% for AFP-L3 at the diagnosis of vascular invasion when cut-off values of 100 ng/mL, 100 mAU/mL, and 15% were adopted, respectively. Although DCP at the time of the initial treatment showed a high ability to predict vascular invasion, DCP at the initial treatment was also positive in only 23 (41.8%) of the 55 patients with a DCP of >100 mAU/mL at the time of vascular invasion development. This result indicates that the tumor characteristics changed during the clinical course.

**Table 3:** Predictors of vascular invasion after RFA (n = 1057).

Variable	Univariate Analysis		Multivariate Analysis	
	HR (95% CI)	*P*	HR (95% CI)	*P*
Age (per 1 year)	1.01 (0.98–1.03)	0.69		
Male sex	0.85 (0.55–1.30)	0.45		
HCVAb-positive	1.10 (0.69–1.75)	0.70		
HBsAg-positive	1.54 (0.93–2.55)	0.11		
Platelet count (per 10^9^/L)	1.00 (0.97–1.04)	0.97		
ALT>80 U/L	0.88 (0.53–1.46)	0.62		
Child Pugh (per 1 point)	1.05 (0.87–1.26)	0.65		
Tumor size (mm)				
≤20	1		1	
21–30	1.54 (0.92–2.57)	0.098	1.30 (0.77–2.19)	0.320
>30	2.51 (1.49–4.22)	<0.001	1.74 (1.01–3.01)	0.048
Tumor number				
1	1		1	
2–3	1.59 (1.04–2.43)	0.033	1.61 (1.05–2.47)	0.029
>3	2.18 (1.13–4.20)	0.002	2.02 (1.05–3.92)	0.037
AFP (ng/mL)				
≤100	1			
101–200	1.41 (0.68–2.94)	0.36		
>200	1.93 (1.18–3.15)	0.008		
DCP (mAU/mL)				
≤100	1		1	
101–200	2.34 (1.23–4.43)	0.008	1.99 (1.03–3.84)	0.041
>200	4.33 (2.62–7.16)	<0.001	3.24 (1.90–5.51)	<0.001
AFP-L3 (%)				
≤10	1		1	
>10	2.22 (1.42–3.48)	<0.001	1.75 (1.10–2.78)	0.018

Abbreviations: HR, hazard ratio; HBsAg, hepatitis B surface antigen; HCVAb, hepatitis C virus antibody; AFP, alpha-fetoprotein; AFP-L3, *Lens culinaris* agglutinin-reactive fraction of AFP; DCP, des-gamma-carboxyprothrombin.

### Treatment of vascular invasion and associated survival outcomes

**Figure 4:**
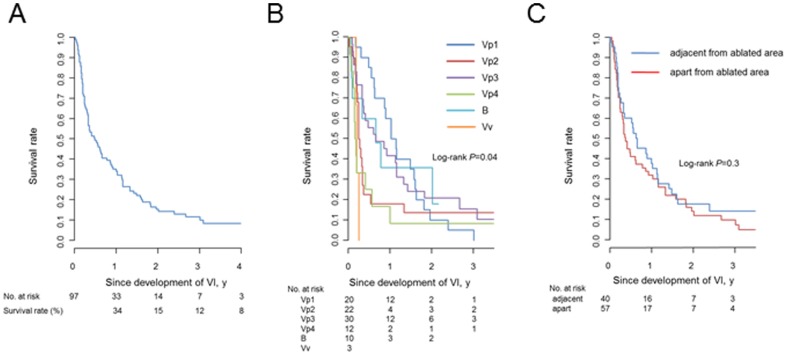
Overall survival rate after development of vascular invasion (A) and survival rate stratified based on severity of invasion (B). Survival rates of patients with vascular invasion developing adjacent to (blue) and apart from (red) the ablated area (C).

Among the 97 patients diagnosed with vascular invasion, 53 (55%) underwent hepatic arterial infusion chemotherapy. Four patients underwent hepatic resection because of a localized tumor in three and tumor shrinkage due to hepatic arterial chemotherapy in one. Six patients received systemic chemotherapy including sorafenib. The remaining patients were treated with combination therapies including TACE, irradiation, and chemotherapy. Twenty-four patients (25%) received supportive therapy due to liver dysfunction or a poor performance status. The 1-, 3-, and 5-year survival rates after development of vascular invasion were 33.1%, 10.6%, and 6.4%, respectively (Fig. 4A). Survival rates differed with the severity of vascular invasion (Fig. 4B), but even in the mildest disease group with Vp1 (invasion within the second branch of the portal vein), survival was still poor (1-, 2-, and 3-year survival rates were 58.9%, 12.0%, and 6.0%, respectively). The survival rate did not change whether the vascular invasion developed adjacent to or apart from the ablated area (Fig. 4C).

## Discussion

Vascular invasion is one of the most important predictors of poor survival of HCC patients [15]–[18]. This study showed that vascular invasion occurred in 10% of patients within 5 years when HCC was initially diagnosed at an early stage. The fact that tumor-related factors at the initial diagnosis could predict the appearance of vascular invasion as a late event may suggest that intrahepatic metastasis of primary tumors can determine overall survival. However, more than half of patients in whom a tumor marker was elevated at the diagnosis of vascular invasion were negative for the tumor marker at the initial diagnosis, which suggests that the appearance of a more aggressive tumor during the clinical course was the direct cause of vascular invasion.

Compatible with our previous report on patients with HCC treated with ethanol injection and microwave ablation, DCP was strongly related to the development of vascular invasion [27]. Some cross-sectional studies reported that DCP was correlated with microvascular invasion [28], [29]. As high DCP tumors were suggested to possess invasive capacity, a high DCP level is proposed to be regarded as a contraindication for liver transplantation in several institutions in Japan [30], [31]. One suggested mechanism behind the relationship between vascular invasion and DCP was that hypoxia in the tumor, which is a key trigger of epithelial-mesenchymal transition, correlates with DCP elevations [32]. It is quite reasonable that tumors with such an invasive phenotype finally develop macrovascular invasion. One concern is that patients with high DCP level may not be suitable for RFA. However, considering the fact that a high DCP level is regarded as a contraindication for liver transplantation and there is no evidence that TACE is superior to RFA in terms of local cure, it would be reasonable to consider resection in patients with good liver function[33] or to perform RFA with a wider margin in unresectable cases with deteriorated liver function when patients show a high DCP level [34].

Several reports have suggested that incomplete thermal ablation might increase tumor aggressiveness. In this study, only 0.66% of ablated nodules developed vascular invasion as local tumor progression [35]. This rate is acceptable considering the low mortality rate related to RFA compared with resection [36], although there is room for technical improvement. In addition, the survival rates of patients with vascular invasion adjacent to and apart from the ablated area were similar. This suggests that vascular invasion might not be a consequence of malignant transformation caused by RFA.

Vascular invasion was diagnosed in an advanced form in 52 of 97 patients, although most of them were followed closely by imaging modalities. One possible reason is that once a tumor invades the vasculature, it extends quite rapidly, probably because there is no obstacle within the lumen. Another reason would be that tumors located in the hilar region of the liver directly invaded the main trunk in some cases. This suggests that early diagnosis of vascular invasion is quite difficult. In addition, it should be noted that even when a minimal extent of portal invasion is diagnosed, the outcome is disappointing with a median survival of 1 year.

After the development of vascular invasion in this study, the therapeutic options were limited and the prognosis was poor. Surgical resection may be preferable in patients with limited tumor extension, but only four patients were indicated for resection; 25% of patients were ineligible for aggressive treatment and received best supportive care because of liver dysfunction caused by the vascular invasion itself or because of repeated recurrence and treatment. Sorafenib is now the treatment of choice for patients with vascular invasion. However, the survival outcome is still unsatisfactory, even in patients with Child-Pugh class A [37], probably because tumors in the portal vein rarely decrease in size with the use of sorafenib and liver function may deteriorate with reduced portal blood flow.

In conclusion, vascular invasion occurs during the clinical course of patients with HCC initially treated with curative intent. The serum DCP level is the most useful predisposing parameter for the development of vascular invasion after RFA. Once vascular invasion has developed, the prognosis is poor. We must develop another strategy by which to improve the survival of these patients.

## Supporting Information

Table S1 **The clinical data of 1,057 patients, which were analyzed in this study.**
(CSV)
